# An unusual cause of ankle pain: fracture of a talocalcaneal coalition as a differential diagnosis in an acute ankle sprain: a case report and literature review

**DOI:** 10.1186/1471-2474-14-111

**Published:** 2013-03-26

**Authors:** Dirk Wähnert, Niklas Grüneweller, Julia Evers, Anna C Sellmeier, Michael J Raschke, Sabine Ochman

**Affiliations:** 1 Department of Trauma, Hand and Reconstructive Surgery, University Hospital Münster, Albert-Schweitzer-Campus 1, Building W1, 48149, Münster, Germany

**Keywords:** Acute ankle sprain, Talocalcaneal coalition, Fracture of coalition, Persisting ankle pain

## Abstract

**Background:**

The acute ankle sprain is one of the most common injuries seen in trauma departments. Ankle sprains have an incidence of about one injury per 10 000 people a day. In contrast tarsal coalition is a rare condition occurring in not more than one percent of the population.

**Case presentation:**

We present the case of a 23 year old male patient with pain and local swelling after an acute ankle sprain. Initial clinical and radiological examination showed no pathologies. Due to prolonged pain, swelling and the inability of the patient to weight bear one week after trauma further diagnostics was performed. Imaging studies (MRI and CT) revealed a fracture of a talocalcaneal coalition. To the knowledge of the authors no fracture of a coalition was reported so far.

**Conclusion:**

This report highlights the presentation of symptomatic coalitions following trauma and furthermore, it points out the difficulties in the diagnosis and treatment of a rare entity after a common injury. A diagnostic algorithm has been developed to ensure not to miss a severe injury.

## Background

The acute ankle sprain is one of the most common injuries seen in trauma departments. Ankle sprains have an incidence of about one injury per 10 000 people a day. In contrast tarsal coalition is a rare condition occurring in not more than one percent of the population. The purpose of the presented case is to describe a 23 year old male patient with pain and local swelling after an acute ankle sprain. Initial clinical and radiological examination showed no pathologies. Due to prolonged pain, swelling and the inability to weight bear further diagnostics revealed a fracture of a talocalcaneal coalition.

## Case presentation

**Figure 1:**
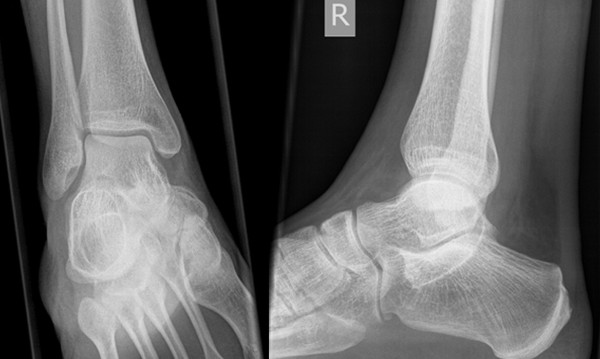
**Conventional x-rays antero-posterior (left) and lateral (right) of the patient after trauma.** No fracture or osseous lesion was found.

A 23 year old male patient arrived at our emergency department after an acute ankle sprain with pain and swelling of the ankle. Injury mechanism was a supination and inversion ankle sprain when dismounting from his bike. The clinical examination showed a swollen right ankle with pressure pain over the lateral malleolus. Examination according to the Ottawa Ankle Rules demonstrated no other local pressure pain. The range of motion was significantly decreased due to the pain. Plain x-ray showed no bony lesions or fractures (Figure 1). The diagnosis of a fibula-calcaneal ligament lesion was posed and an ankle orthosis was applied. The patient was discharged mobilized with crutches under pain adapted weight bearing and heparin for thrombosis prophylaxis. Additionally he got analgic drugs.

After one week the patient returned to our hospital. He reported to be unable to weight bear and to have severe pain. The clinical examination showed a hematoma around the lateral malleolus.

**Figure 2:**
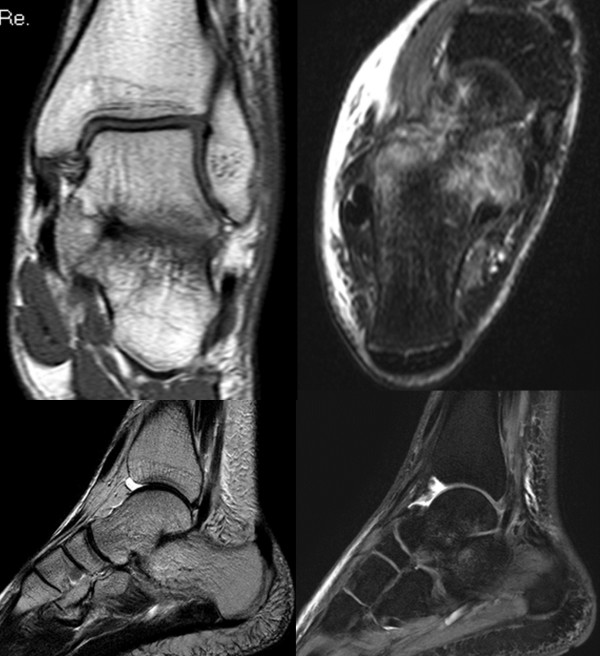
Several MRI slices showing the lateral ligament injury (left upper), the bone bruise in the calcaneus (right upper) and the coalition in the lower pictures.

An MRI scan was performed. The scan showed a rupture of the fibulo-calcaneal and the tibio-calcaneal ligament as well as bone bruise between talus and calcaneus. Additionally a line of the medial talar facet was found (Figure 2). Due to this finding we performed a CT scan of the right foot.

**Figure 3:**
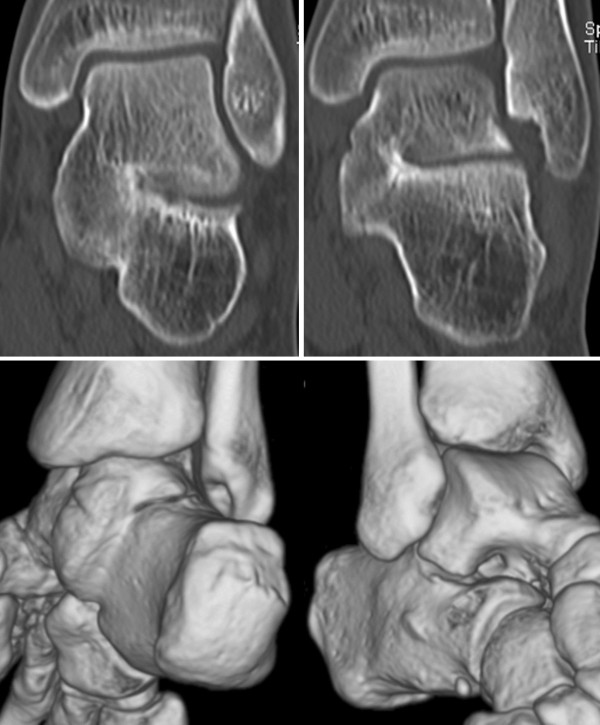
Two coronar slices from the CT scan showing the osseous talocalcaneal coalition with the fracture line (upper part), the lower part shows the coalition in the 3D reconstruction.

The CT scan showed a fracture of a taloclcaneal coalition. This bony coalition was located between the medial talar facet and the sustentaculum of the calcaneus (Figure 3).

After this diagnosis we changed the therapeutical strategy to an immobilising lower leg orthesis. The patient was admitted to full weight bear in respect to his pain. The pain medication was addapted and thrombosis phrophylaxis continued.

## Conclusion

### Epidemiology

Tarsal coalition is an uncommon disorder with bony, cartilaginous or fibrous union between two or more bones of the hind- and midfoot.

The accepted theory for the etiology of tarsal coalitions is a failure of complete segmentation of the mesenchyme with the absence of normal joint formation during embryonic period [1,2]. An autosomal dominant inheritance pattern has been suggested [1]. Some reasons for acquired coalitions are e. g. clubfoot deformities, inflammatory arthritis, osteoarthritis, intra-articular fractures, osteonecrosis and malignancies [2].

The tarsal coalition is a rare condition occurring in not more than one percent of the population [1,3]. However, the true prevalence of tarsal coalitions is unknown. All clinical studies miss the asymptomatic coalitions. Leonard even found 76% of subjects with tarsal coalition as symptom free [1]. A recent prospective MRI study showed a 12% prevalence of tarsal coalition [4].

According to current data half of the patients with a tarsal coalition show bilateral appearance. There is a slight male predominance.

With together about 90° the most frequent observed coalitions are the calcaneonavicular and talocalcaneal. Due to the easy recognition of the calcaneonavicular coalition on plane radiographs, in the past, this coalition was thought to be the most common. However, CT and MRI have shown nearly equal incidences for this both coalitions.

In contrast the acute ankle sprain is one of the most common injuries in trauma departments. Ankle sprains have an incidence of about one injury per 10 000 people a day. With an incidence of 52.7 per 10 000 people per year lesions of the lateral ligament complex, due to an ankle sprain, are the most common injuries of the human body [5-8]. An investigation of Suhr et al. showed the source of injury in 416 acute ankle sprains. 37% of the sprains were caused during sports activities, 33% happened in off time and 30% occurred at work. Sports with a high risk for ankle sprains are: football, volleyball, basketball, rugby, tennis and athletic [8].

### Biomechanics

Depending on the location of the coalition the gait cycle is more or less affected. The triple complex responds to the rotatory motion of the tibia on the foot during gait cycle. The subtalar joint and transverse tarsal joints (talonavicular and calcaneonavicular) allow the foot to change from a flexible shock absorber to a rigid lever arm [2]. Therefore, increasing rigidity at the subtalar joint results in dysfunctional shock absorption during gait [9]. The talonavicular joint is the most important for the mobility of the triple joint complex causing severe problems in patients with tarsal coalitions.

Coalitions involving two or more joints can lead to excessive stresses in the hindfoot causing pain, inflammation and premature joint degeneration.

The functional absent subtalar joint causes stress concentration at the ankle joint with leading symptoms such as ankle sprain and ankle pain.

The majority of ankle sprains (85%) are supination events (combined plantar flexion, adduction, and inversion), pronation (combined dorsiflexion, abduction, and eversion) accounts for 15% [10]. Up to 40% of the patients develop persisting disorders like chronic pain or chronic ligamentous instability with recurrent sprains even after minor trauma [8,10,11]. Krause et al. found inhomogeneous load distribution in the unstable hindfoot as well as intra-articular pressure elevation in the ankle and subtalar joint during hindfoot supination sprains with intact ligaments or incompetent ligaments in a biomechanical investigation. They conclude, that these circumstances are substantial for the development of osteochondral lesions with all their follow up [10].

Fracture occurs in less than 15% of all ankle sprains [12].

### Clinic

The patient in our case presented in our emergency department with the typical symptoms after acute ankle sprain: immobilizing pain (he was not able to weight bear at all), swelling around the ankle and lateral hematoma around the malleolus. He reported to have had no symptoms or limitations prior to the trauma.

The symptoms related to tarsal coalitions are variable. The onset of symptoms is related to the progression of the ossification [9]. This may be a result of repetitive biomechanical stress through physical activity. Repeated micro fractures and remodeling cause progression of the ossification in the coalition. This increases rigidity and clinical symptoms [9].

Generally symptoms appear in the second decade of life [13]. The majority of patients with tarsal coalitions reaching their 20s without symptoms will never develop symptoms [2]. Most patients are represented with hindfoot pain, a limited range of motion (unilateral coalition) or stiffness. The pain is often prominent and located around the ankle mainly on the lateral and antero-lateral aspect of the ankle. Symptoms often appear after recurrent trauma (ankle sprain) or increasing athletic activity [2,9,14].

There are only a few reports of fractures involving tarsal coalitions. Kim et al. reported the case of a 15 year old cross-country runner who fell and sustained an axial load to his heel. The x-rays showed an intraarticular calcaneus fracture; the talocalcaneal coalition was not clearly seen. CT-scan showed a middle facet coalition bilaterally. Prior to the accident the patient denied any pain or limitations. The calcaneus fracture was treated operatively by plate and screw osteosynthesis. The patient returned to full activity [15]. Moe et al. described the case of a 48 year old woman presenting with heel pain which persisted for 3 month. There was no accident in the history described. The diagnostic showed a posterior talocalcaneal coalition with an oblique calcaneal stress fracture [13].

### Diagnostic

After clinical examination conventional radiographs in two planes (antero-posterior and lateral) of the ankle are a first line standard diagnostic tool after ankle injury (following the Ottawa ankle rules [16]) to exclude a fracture. After acute ankle sprain a second clinical examination few days after the injury is recommended to distinguish a ligament rupture from a simple sprain.

In the diagnosis of tarsal coalition conventional radiographs in two planes are often sufficient to diagnose most calcaneonavicular and talonavicular coalitions [17].

In contrast, talocalcaneal coalitions generally require cross-sectional imaging for confirmation and characterization. Talocalcaneal coalitions are difficult to visualize on standard radiographs due to the complex three-dimensional orientation of the subtalar joint. There have been a number of *secondary radiographic signs* described to diagnose talocalcaneal coalitions (Table 1). These findings develop secondary to the coalition because of the alteration in hindfoot biomechanics [17].

**Table 1:** Radiological characteristics of talar coalitions

**coalition**	**x-ray**	**CT**	**MRI**
***calcaneonavicular***	osseous bridging	not necessary	not necessary
***talonavicular***	osseous bridging	not necessary	not necessary
***talocalcaneal***	*- talar beak* navicular overriding the talus	coronal and axial planes	T1 and T2 sequences
		*- osseous coalitions* bony bridging	- bony coalition
	*- C sign* bony bridging between talus and calcaneal sustentaculum		
			- fibrous coalition
			- cartilaginous coalition
		- *non-osseous coalitions* facet narrowing, reactive changes (e. g. cysts, hypertrophy), downward slope or broadening of sustentaculum	fat suppressed sequence (STIR)
	*- narrowing of the posterior subtalar joint*		
			- bone marrow edema (fracture)
	*- rounding of the lateral talar process*		
	*- lack of depiction of the middle facets on the lateral radiograph*		
	*- short talar neck*		
	*- dysmorphic sustentaculum tali*		
	*Combination of signs sensitivity/specifity 100 %/ 88 %*		

**Figure 4:**
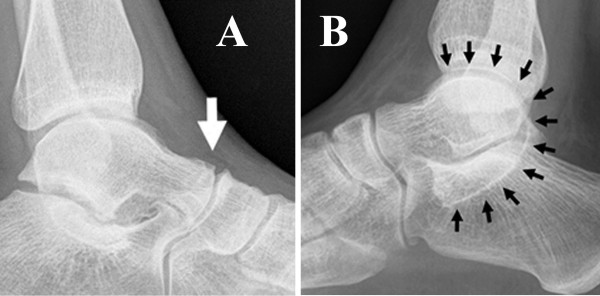
(A) lateral radiograph of a left ankle showing the talar beak (arrow), (B) lateral radiograph of the right ankle obtained of our patient with C sign (arrows) extending from the talar dome through the coalition component of the posterior talocalcaneal joint to the sustentaculum tali.

The *talar beak* (Figure 4A) can occur due to the decreased subtalar joint motion. This results in the navicular overriding the talus. The mechanism is a periosteal elevation at the insertion of the talonavicular ligament with secondary osseous repair.

Another radiographic sign is the so called *C sign* (Figure 4B), which was first described by Lateur et al. in 1994 [14]: a c-shaped line formed by the outline of the medial talar dome and the inferior outline of the sustentaculum tali. This sign results from bony bridging between the talar bone and the calcaneal sustentaculum, as well as the inferior outline of the sustentaculum. Lateur et al. reported a sensitivity and specificity of 87% and 93%, respectively [14,18]. Sakellariou et al. reported a sensitivity of the C sign of 98% [19]. Taniguchi et al. re-evaluated the C sign for the diagnostic of talocalcaneal coalitions on 110 lateral radiographs (55 with and 55 without coalition). Two observers assessed the presence of the C sign. They found an overall sensitivity and specificity of 49% and 91%, respectively. This group also found an age and coalition type related sensitivity. For patients younger than 12 years the sensitivity was 5%, between 13 and 20 years it was 80% and over 21 years sensitivity was 70%. For the medial type sensitivity was 66%, the posterior type showed no C sign (sensitivity 0%) and 100% of diffuse coalitions showed the C sign. These circumstances put the C sign as diagnostic radiographic sign into perspective and illustrate the advantage of cross-sectional imaging for subtalar coalitions.

Additional radiographic signs described in the literature are the *narrowing of the posterior subtalar joint, rounding of the lateral talar process, lack of depiction of the middle facets on the lateral radiograph, a short talar neck and a dysmorphic sustentaculum tali.*

Crim et al. retrospectively investigated the combination of the above described radiographic signs in the diagnosis of talar coalitions. They found a sensitivity and specificity of 100% and 88%, respectively, when combining the sings for diagnostic of a talocalcaneal coalition [20].

CT scan of the ankle and hindfoot should be performed in coronal and axial planes. Thus, coalitions of all types are usually easily detected. Computer tomography is essential in the diagnosis of talocalcaneal coalition and also in fractures associated with coalitions. Talocalcaneal coalitions are best visualized on coronal planes. In osseous coalitions bony bridging can be found in the CT scan. In non-osseous coalitions facet narrowing, reactive changes of the underlying bone (e. g. cysts, hypertrophy), downward slope of the sustentaculum or broadening of the sustentaculum can be the only changes visible. An anatomical dissection and computer tomography study of Solomon et al. investigated the epidemiology and diagnostic power of CT scan for tarsal coalitions in 100 cadaver feet [21]. They found nine non-osseous talar coalitions (two talocalcanar, seven calcaneonavicular). The CT diagnosed one osseous talocalcanear coalition and was suspicious of eight non-osseous coalitions. The CT scan diagnosed 55.5% of the coalitions. However, CT did not diagnose four non-osseous coalitions and diagnosed four coalitions by mistake. Solomon et al. conclude that CT has a low sensitivity in the detection of non-osseous coalitions; they recommend not using CT routinely in the diagnoses of tarsal coalitions.

MR imaging of tarsal coalitions is another diagnostic method, which should be performed including T1 and T2-weighted sequences, additionally a fat suppressed sequence (short-inversion-time inversion recovery – STIR) is recommended to identify bone marrow edema (fractures) and soft-tissue edema or inflammation. The MRI can determine the density of the bridging material and thus, can differentiate between bony and fibrous or cartilaginous coalitions. In complete osseous coalition bone marrow is visible across the fused articulation. In non-osseous coalition joint space is reduced, additionally in cartilaginous coalition a cartilage or fluid iso-intens area may be present. For fibrous coalition low-signal intensity in the affected joint can be characteristic. The T1- and T2-weighted fat saturated as well as the STIR sequence can show fractures in terms of bone marrow and periosteal edema. The STIR sequence also regularly shows bone marrow edema along the fused articulation.

CT or MRI? Wechsler et al. compared preoperative MRI and CT scans with intraoperative results (9 tarsal coalitions and one synovitis). CT depicted six coalitions of which four were characterized correctly, whilst fibrous coalitions were not characterized correctly. MRI depicted all coalitions (seven correctly characterized), but a proliferative synovitis was incorrectly characterized as a fibrous coalition [22]. Emery et al. compared CT and MRI scans of twenty patients presenting with symptoms of tarsal coalitions. Both MRI and CT missed one coalition. They conclude that MRI can be performed and provide nearly equivalent diagnostic accuracy for detecting tarsal coalition compared to the gold standard CT [23].

### Classification

Anatomically ankle sprains can be classified by the affected ligaments [24]:

Tarsal coalitions are first classified by the involved bones (e.g. talonavicular, talocalcnear, calcaneonavicular). Additionally the morphology of bridging is classified as osseous or non-osseous. The non-osseous coalitions can be differentiated into fibrous or cartilaginous.

**Figure 5:**
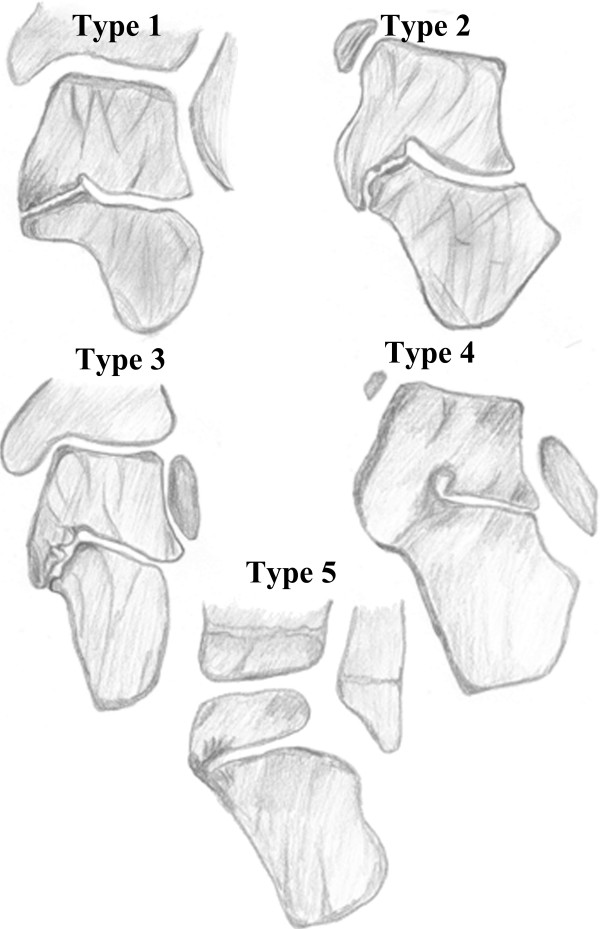
**Classification of talocalcaneal coalitions based on 3D reconstruction of computer tomography by Rozansky et al. [**[25]**]. *Type 1****– linear*: fibrocartilaginous linear coalition parallel to the subtalar joint; ***Type 2****– linear with posterior hook*: fibrocartilaginous coalition linear anteriorly, which curves into a posterior hook overlapping the sustentaculum tali medial and dorsal; ***Type 3****– shingled*: coalition with an orientation that sloped down in an overlapping fashion, with the talar portion shingled over the calcaneal; ***Type 4****– complete osseous*: complete osseous coalition of the medial facet; ***Type 5****– posterior*: small, peripheral posterior coalition.

Rozansky et al. developed a classification of talocalcaneal coalitions based on 3D CT reconstructions (Figure 5). They used 54 coalitions to put them into five types [25].

### Treatment

The treatment of the acute ankle sprain is conservative in first line. Options include the use of ice and compression, in combination with rest and elevation in the acute phase. Functional treatment for 4 to 6 weeks is preferable to immobilization. Nevertheless, a short period (10 days) of plaster immobilization facilitating a rapid decrease of pain and swelling and can therefore be helpful in the acute phase. For stabilization the use of a semirigid brace is recommended. Additional exercise therapy should be used [11].

The surgical treatment can be considered after failed conservative treatment (e.g. persisting instability, pain) [11]. In an investigation of Suhr et al. 15.9% of patients underwent surgery after primary conservative treatment after an ankle sprain within one year after trauma [8].

The first step in the treatment of tarsal coalitions must be the conservative therapy. This includes hard soled shoes and foot and/or ankle stabilizing orthoses. Inflammation can be treated by oral non-steroidal anti-inflammatory drugs. Immobilizing for a period of 6 weeks is yet another possibility.

If the conservative treatment fails surgical intervention can be recommended. The two most prominent methods are the excision of the coalition and the arthrodesis of the involved joints.

For a fracture of the coalition no recommendation in the literature can be found. Kim et al. reported a case of a calcaneus fracture in a 15-year old cross-country runner. Due to the fracture displacement and the decreased Boehler’s angle they did an open reduction and internal fixation [15]. In contrast, Moe et al. treated a non-displaced calcaneal stress fracture conservatively with partial weight bearing.

In our case we decided to choose a conservative treatment, too. The fracture was not displaced. We treated the patient using an immobilizing orthosis allowing the patient pain adapted weight bearing.

### Summary

**Figure 6:**
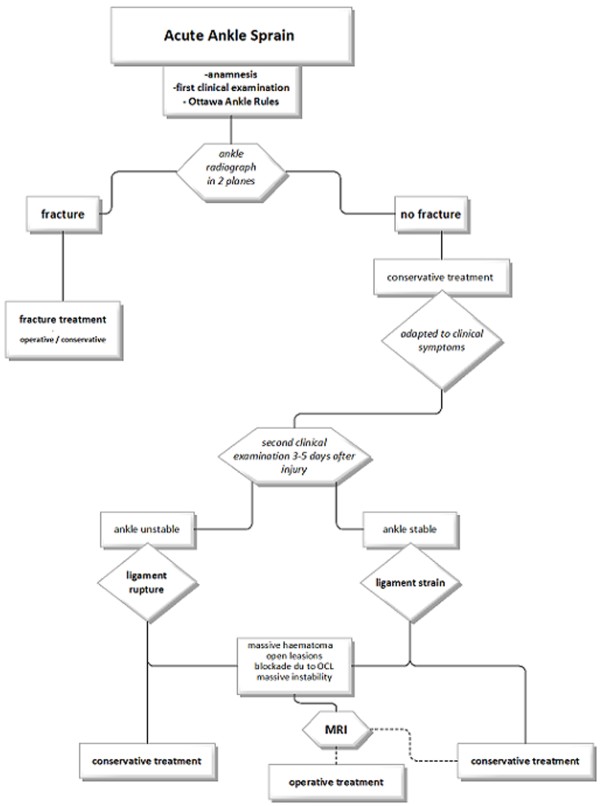
Diagnostic algorithm of the acute ankle sprain with stepwise diagnostic escalation.

Tarsal coalitions are rare entities in the daily routine of trauma and orthopedic surgeons. Even more uncommon is the fracture of a coalition. Nevertheless, the reported case should point out the advantage of an established stepwise diagnostic procedure. This ensures that no injuries will be missed and optimal therapy for the patient is given (Figure 6).

## Consent

Written informed consent was obtained from the patient for publication of this Case report and any accompanying images. A copy of the written consent is available for review by the Editor of this journal.

## Competing interests

The authors declare that they have no competing interests.

## Authors’ contributions

DW was involved in drafting the manuscript and preparing the figures; NG and JE performed the clinical investigation and contributed in the appropriate sections to the manuscript. ACS and MJR did the drawings and assisted writing the Review section. SO was responsible to create the algorithm and in writing the manuscript. All authors read and approved the final manuscript. All authors were involved in the final approval of the submitted version.

## Authors’ information

DW, NG, JE and ACS are residents at the Department of Trauma, Hand and Reconstructive Surgery of the University Hospital Münster, they are clinically and scientifically mainly focused on foot and ankle surgery, MJR is the head of the Department, SO is the head of the Foot and Ankle Group.

## Pre-publication history

The pre-publication history for this paper can be accessed here:

http://www.biomedcentral.com/1471-2474/14/111/prepub

## References

[B1] Leonard MA The inheritance of tarsal coalition and its relationship to spastic flat foot J Bone Joint Surg Br 1974 56B 3 520 526 4421359

[B2] Bohne WH Tarsal coalition Curr Opin Pediatr 2001 13 1 29 35 10.1097/00008480-200102000-00005 11176240

[B3] Snyder RB Lipscomb AB Johnston RK The relationship of tarsal coalitions to ankle sprains in athletes Am J Sports Med 1981 9 5 313 317 10.1177/036354658100900505 6792935

[B4] Nalaboff KM Schweitzer ME MRI of tarsal coalition: frequency, distribution, and innovative signs Bull NYU Hosp Jt Dis 2008 66 1 14 21 18333823

[B5] Hintermann B Biomechanics of the unstable ankle joint and clinical implications Med Sci Sports Exerc 1999 31 7 Suppl S459 S469 10416547 10.1097/00005768-199907001-00007

[B6] Mack RP Ankle injuries in athletics Clin Sports Med 1982 1 1 71 84 7186843

[B7] Bridgman SA Clement D Downing A Walley G Phair I Maffulli N Population based epidemiology of ankle sprains attending accident and emergency units in the West Midlands of England, and a survey of UK practice for severe ankle sprains Emerg Med J 2003 20 6 508 510 10.1136/emj.20.6.508 14623833 PMC1726220

[B8] Suhr A Muckley T Hofmann GO Spahn G Therapy of acute ankle sprain: one-year results of primary conservative treatmen Sportverletz Sportschaden 2012 26 1 39 44 22422283 10.1055/s-0031-1299108

[B9] Schenkel D Degraauw J Degraauw C Talocalcaneal coalition in a 15 year old female basketball player J Can Chiropr Assoc 2010 54 4 222 228 21120013 PMC2989394

[B10] Krause F Blatter S Waehnert D Windolf M Weber M Hindfoot joint pressure in supination sprains Am J Sports Med 2012 40 4 902 908 10.1177/0363546511432550 22238054

[B11] Kerkhoffs GM van den Bekerom M Elders LA van Beek PA Hullegie WA Bloemers GM Diagnosis, treatment and prevention of ankle sprains: an evidence-based clinical guideline Br J Sports Med 2012 10.1136/bjsports-2011-090490 22522586

[B12] Bachmann LM Kolb E Koller MT Steurer J Riet G Accuracy of Ottawa ankle rules to exclude fractures of the ankle and mid-foot: systematic review BMJ 2003 326 7386 417 10.1136/bmj.326.7386.417 12595378 PMC149439

[B13] Moe DC Choi JJ Davis KW Posterior subtalar facet coalition with calcaneal stress fracture AJR Am J Roentgenol 2006 186 1 259 264 10.2214/AJR.04.1614 16357413

[B14] Lateur LM Van Hoe LR Van Ghillewe KV Gryspeerdt SS Baert AL Dereymaeker GE Subtalar coalition: diagnosis with the C sign on lateral radiographs of the ankle Radiology 1994 193 3 847 851 7972836 10.1148/radiology.193.3.7972836

[B15] Kim DH Berkowitz MJ Fracture of the calcaneus associated with talocalcaneal coalition Foot Ankle Int 2004 25 6 426 428 15215030 10.1177/107110070402500612

[B16] Stiell I Ottawa ankle rules Can Fam Physician 1996 42 478 480 8616287 PMC2146318

[B17] Newman JS Newberg AH Congenital tarsal coalition: multimodality evaluation with emphasis on CT and MR imaging Radiographics 2000 20 2 321 332 quiz 526–7, 532 10715334 10.1148/radiographics.20.2.g00mc03321

[B18] Kim SH The C, sign Radiology 2002 223 3 756 757 10.1148/radiol.2233991817 12034945

[B19] Sakellariou A Sallomi D Janzen DL Munk PL Claridge RJ Kiri VA Talocalcaneal coalition. Diagnosis with the C-sign on lateral radiographs of the ankle J Bone Joint Surg Br 2000 82 4 574 578 10.1302/0301-620X.82B4.10263 10855886

[B20] Crim JR Kjeldsberg KM Radiographic diagnosis of tarsal coalition AJR Am J Roentgenol 2004 182 2 323 328 10.2214/ajr.182.2.1820323 14736655

[B21] Solomon LB Ruhli FJ Taylor J Ferris L Pope R Henneberg M A dissection and computer tomograph study of tarsal coalitions in 100 cadaver feet J Orthop Res 2003 21 2 352 358 10.1016/S0736-0266(02)00131-6 12568969

[B22] Wechsler RJ Schweitzer ME Deely DM Horn BD Pizzutillo PD Tarsal coalition: depiction and characterization with CT and MR imaging Radiology 1994 193 2 447 452 7972761 10.1148/radiology.193.2.7972761

[B23] Emery KH Bisset GS 3rd Johnson ND Nunan PJ Tarsal coalition: a blinded comparison of MRI and CT Pediatr Radiol 1998 28 8 612 616 10.1007/s002470050430 9716636

[B24] de Cesar PC Avila EM de Abreu MR Comparison of magnetic resonance imaging to physical examination for syndesmotic injury after lateral ankle sprain Foot Ankle Int 2011 32 12 1110 1114 10.3113/FAI.2011.1110 22381194

[B25] Rozansky A Varley E Moor M Wenger DR Mubarak SJ A radiologic classification of talocalcaneal coalitions based on 3D reconstruction J Child Orthop 2010 4 2 129 135 10.1007/s11832-009-0224-3 20234768 PMC2832879

